# Apoptotic markers and Dectin-1^+^ cells predict cardiac function in patients with ST-elevation myocardial infarction after percutaneous coronary intervention: a retrospective cohort study

**DOI:** 10.1186/s12872-026-05988-2

**Published:** 2026-05-21

**Authors:** Fengjing Yang, Bingjun Shu, Na Kuang, Jing Qiu, Jing Wang, Jing Liu

**Affiliations:** 1 Department of Cardiovascular Medicine, Wuhan Hospital Of Traditional Chinese Medicine, Wuhan, 430000 China; 2 Department of Medical Records and Statistics, Wuhan Hospital Of Traditional Chinese Medicine, Wuhan, 430000 China; 3 Department of Acupuncture, Wuhan Hospital Of Traditional Chinese Medicine, Wuhan, 430000 China

**Keywords:** Apoptotic Markers, Dectin-1 + Cells, ST-Elevation Myocardial Infarction, Percutaneous Coronary Intervention, Left ventricular ejection fraction

## Abstract

**Background:**

To investigate the prognostic value of apoptotic markers and dendritic cell-associated C-type lectin-1 (Dectin-1)-positive cells on cardiac function in patients with ST-elevation myocardial infarction (STEMI) after percutaneous coronary intervention (PCI).

**Methods:**

Patients with STEMI who underwent successful PCI at Wuhan Hospital of Traditional Chinese Medicine from March 2019 to June 2022 were retrospectively analyzed. Patients were divided into two groups based on whether left ventricular ejection fraction (LVEF) improved < 10% (the LVEF non-improvement group) or ≥ 10% (the LVEF improvement group) at 30 days post-PCI compared to that at 24 h after PCI. Blood test results, imaging measurements, apoptotic markers, and soluble Dectin-1 level were compared between the two groups. Multivariable logistic regression was performed adjusting for age, sex, diabetes, and LAD occlusion rate.

**Results:**

Among 96 patients included, 28 showed LVEF improvement. Compared to that in the LVEF non-improvement group, the expression levels of tumor necrosis factor-like weak inducer of apoptosis (TWEAK) and Dectin-1 were significantly lower in the LVEF improvement group (*P* < 0.05), while the expression level of tumor necrosis factor-related apoptosis-inducing ligand (TRAIL) was significantly higher in the LVEF improvement group (*P* < 0.05). TRAIL (*P* = 0.047) and TWEAK (*P* = 0.005) were independently associated with LVEF improvement after adjusting for confounders. TRAIL, TWEAK, soluble Dectin-1, and a combination of these three biomarkers exhibited prediction sensitivity for LVEF improvement in post-PCI patients with STEMI of 50.00%, 60.71%, 67.86%, 78.57%, respectively, and specificity of 79.41%, 73.53%, 57.35%, 82.35%, respectively.

**Conclusions:**

Apoptotic markers and soluble Dectin-1 may serve as potential predictors of cardiac function recovery in patients with STEMI following PCI, and the combination of these markers showed improved prognostic value, though further validation in larger, prospective cohorts is warranted.

## Background

Despite advancements in interventional therapies, acute myocardial infarction (AMI), a critical manifestation of coronary heart disease, is still a life-threatening condition with a high mortality rate [1, 2]. Although percutaneous coronary intervention (PCI) has significantly improved the survival rates among patients with ST-elevation myocardial infarction (STEMI), post-PCI cardiac function impairment with an elevated risk of heart failure and death, remains a persistent concern [3]. It is essential to identify robust prognostic indicators so as to enhance clinical prognosis in such patients. Hence, accurate prediction of risk of post-PCI heart failure in patients with STEMI holds significant clinical importance.

Cardiomyocyte apoptosis, a hallmark of myocardial infarction, plays a pivotal role in the progression of heart failure [4, 5]. Thus, apoptotic biomarkers hold promise as predictive tools for the assessment of cardiac function following AMI. Tumor necrosis factor-like weak inducer of apoptosis (TWEAK), a multifunctional cytokine in the tumor necrosis factor (TNF) family, is widely expressed across various tissues including heart tissue and is involved in the regulation of critical cellular processes such as growth, migration, differentiation, and survival, all of which are intricately linked to pathological cardiovascular remodeling [6, 7]. Tumor necrosis factor-related apoptosis-inducing ligand (TRAIL) has been implicated in post-infarction myocardial injury by binding to its receptor, which activates caspase 8, subsequently triggering caspase 3 and the downstream apoptotic pathway [8]. TRAIL elicits diverse cellular responses including activation, proliferation, differentiation, and apoptosis through its receptor interactions [9]. Dendritic cell-associated C-type lectin-1 (Dectin-1), a member of the C-type lectin-like receptors (CLRs), is predominantly expressed on the surface of activated myeloid cells [10]. Dectin-1 is involved in the regulation of a variety of cellular responses, including cellular phagocytosis, autophagy, respiratory burst, and production of inflammatory factors [11], and plays an important role in inflammation, cardiac remodeling and dysfunction [12, 13]. In addition, emerging evidence suggested a potential association between Dectin-1^+^ cells and post-infarction heart failure [14]. However, such relationships are rarely explored in clinical studies.

Most of the existing predictive models in post-PCI patients with STEMI focus on the prediction of major adverse cardiovascular events [15, 16] or in-hospital mortality [17]. There is a lack of prediction models for improvement of left ventricular ejection fraction (LVEF). In a previous study of prediction of left ventricular systolic dysfunction, only clinical parameters were considered in the model [18], while apoptotic biomarkers were not included.

In this study, we aimed to explore the predictive value of apoptosis biomarkers of TWEAK and TRAIL as well as Dectin-1^+^ cells in the improvement of LVEF in patients with STEMI after PCI.

## Methods

### Study population

From March 2019 to June 2022, patients who were aged ≥ 18 years and received successful PCI after diagnosis with STEMI at the Department of Cardiology, Wuhan Hospital of Traditional Chinese Medicine, were retrospectively included in the study. Patients with incomplete clinical record were excluded. Patients with active infection, immune disorders, or malignant tumors were also excluded.

Patients were required to meet the diagnostic criteria of STEMI as follows:

1. Typical ischemic chest pain consistent with STEMI: severe crushing pain in the retrosternal or precordial area persisting for more than 30 minutes, potentially radiating to the left upper arm, jaw, neck, back or shoulder. Associated symptoms may include nausea, vomiting, profuse sweating, respiratory distress, and in some cases, syncope. The symptoms cannot be completely relieved by nitroglycerin.
2. Dynamic electrocardiographic changes: The hallmark electrocardiographic feature of STEMI is ST-segment elevation, in the form of bow-back and upward, with or without pathologic Q-wave and R-wave decompensation. In the early stage of STEMI, there is a change in the hyperacute T-wave (abnormally large and asymmetric in the two branches) and/or ST-segment elevation in the form of a tilt-straight pattern. These changes progress into a fusion of the ST and T segments, and accompanied by a mirrored ST-segment depression in the corresponding leads.
3. Serological and imaging examinations: Patients whose symptoms and ECG allow a definitive diagnosis of STEMI do not need to wait for myocardial injury markers and/or imaging results. Myocardial injury marker levels, preferably cTn, should be routinely measured in the acute phase, but this should not delay reperfusion therapy, and it is advisable to dynamically observe the evolution of myocardial injury markers. Imaging examinations such as echocardiography can help in the differential diagnosis and risk stratification of patients with acute chest pain.
4. Coronary angiography confirming amenability of patient's infarct-related vessels to PCI.

LVEF was calculated by subtracting the end-systolic left ventricular (LV) volume from the end-diastolic LV volume and dividing it by the end-diastolic LV volume in echocardiogram. To limit the variation, LVEF was measured by modifed Simpson and confirmed by two experienced experts. All patients were divided into two groups according to whether LVEF improved < 10% (the LVEF non-improvement group) or ≥ 10% (the LVEF improvement group) at 30 days post-PCI compared to that at 24 h.

The study was approved by the Ethics Committee of Wuhan Hospital of Traditional Chinese Medicine. Written informed consents were waived due to the retrospective nature of this study.

### Coronary angiography (CAG)/PCI

Patients underwent CAG immediately following diagnosis of STEMI by using a digital subtraction machine (Artiszee AXIOM; Siemens Ltd, Erlangen, Germany) in the catheterization laboratory according to standard procedures. Patients were placed in the supine position. After routine disinfection, toweling and local anesthesia, a catheter was introduced via a puncture artery to assess both the right and left coronary arteries. CAG was performed from several angles, including two positions in the right coronary artery and four positions in the left coronary artery. Additional positions were further obtained if necessary. Two cardiologists independently assess the coronary stenosis and were blinded to patient information. The stenosis percentage was calculated as ratio of the internal diameter of the stenosis to the internal diameter of the proximal normal coronary artery.

PCI procedures were performed by experienced cardiovascular physicians. All patients received oral administration of aspirin 300 mg and ticagrelor 180 mg, and promptly underwent CAG to identify the offending vessel. The vessel patency rate was 100%.

### Baseline characteristics and blood sample collection

Age, sex, history of hypertension, diabetes mellitus, smoking status, and history of coronary heart disease were collected. Coronary angiographic data including culprit vessel location, number of vessels involved, initial TIMI flow grade, post-PCI TIMI flow grade, and coronary artery occlusion rates were recorded. Time from symptom onset to vessel recanalization was documented. Venous blood was drawn upon admission, including routine blood tests, blood urea nitrogen (BUN), creatinine (Cr), uric acid (UA), cholesterol (TC), low-density lipoprotein cholesterol (LDL-C), triglycerides (TG), cardiac troponin I (cTNI), myoglobin, creatine kinase-MB (CK-MB), human brain natriuretic peptide (BNP), D-dimer, fibrinogen, white blood cell (WBC) count, platelet (PLT) count, hypersensitive C-reactive protein (hs-CRP), alanine aminotransferase (ALT), and aspartate aminotransferase (AST). Additionally, the venous blood was centrifuged at 3000 revolutions per minute for 10 min at 4 °C. The separated serum was stored at -80 °C until analysis of serum apoptotic markers TWEAK and TRAIL as well as soluble Dectin-1 at required timepoints.

### Enzyme-linked immunosorbent assay (ELISA)

Soluble Dectin-1 and serum apoptosis markers (including TWEAK and TRAIL) were assessed using ELISA kits (Item No. HM12599, HM11178, and HM10365; Bioswamp). The ELISA kits were allowed to equilibrate at room temperature for 30 min, following which standards were appropriately diluted according to the provided instructions. Serum samples were thawed at room temperature for 30 min, then added to the designated wells, followed by thorough mixing, washing, coloration, and termination according to the manufacturer’s protocol. Subsequently, the absorbance (OD) of each well was measured sequentially at 450 nm, with readings zeroed using a blank well. The quadratic regression equation of the standard curve was calculated using the OD value of the standard as the horizontal coordinate and the concentration as the vertical coordinate. The OD value of the sample was then substituted into this equation to determine the sample concentration, thereby obtaining the actual concentration of the sample.

### Follow-up

All patients were followed up in the outpatient clinic 30 days after PCI. LVEF and other examinations were performed. Patients were further followed for at least one year for major adverse cardiovascular events (MACE) either by clinical visit or by telephone. MACE were defined as endpoints of cardiac death, myocardial reinfarction, malignant arrhythmia, and acute heart failure.

### Statistical analysis

Data analysis was performed using SPSS 20.0 (IBM Corp., Armonk, NY, USA). Continuous variables with normal distribution were described using mean ± standard deviation, while those with non-normal distribution were described using median (interquartile range). Categorical variables were presented as numbers and percentages. Patients were categorized into two groups based on improvement of ≥ 10% or < 10% in LVEF at 30 days post-PCI compared to that at 24 h post-PCI. Between-group comparisons of continuous variables were performed using independent samples t-test, while categorical variables were analyzed using the chi-square test or exact probability method. Multivariable logistic regression analysis was performed adjusting for age, sex, diabetes, and LAD occlusion rate. A subgroup analysis was further performed in patients with baseline LVEF ≥ 50% to address the concern of regression-to-the-mean. Pearson correlation analysis was used to determine the correlations among serum levels of soluble Dectin-1, TRAIL, and TWEAK. Receiver operating characteristic (ROC) curves for ≥ 10% improvement in LVEF were generated for TRAIL, soluble Dectin-1, and TWEAK, respectively. Area under the curves (AUCs) and optimal prediction cutoffs of each individual biomarker were first determined and converted to dichotomous variables. The odds ratios (ORs) of these dichotomous variables were then calculated using logistic regression. The ROC curve was also generated for combining TRAIL, soluble Dectin-1, and TWEAK. The AUC, sensitivity, and specificity of combining all three biomarkers were further explored. *P* < 0.05 was considered statistically significant.

## Results

### Baseline characteristics

Between March, 2019 to June, 2022, a total of 96 patients were included, with 28 in the LVEF improvement group and 68 in the LVEF non-improvement group. There were 76 male patients and 20 female patients. The mean age was 60.04 ± 12.53 years. LVEF improvement was not associated with age, sex, cTNI, myoglobin, CK-MB, BNP, Cr, UA, TC, TG, LDL-C, ALT, AST, D-dimer, fibrinogen, WBC count, PLT count, or hs-CRP (all *p* > 0.05, Table 1). Diabetes was more prevalent in the LVEF improvement group (39.3% vs. 17.6%, *p* = 0.024, Table 1). The LVEF at baseline was significantly lower in the LVEF improvement group than that in the non-improvement group (48.21 ± 7.83 vs. 60.25 ± 5.81, *p* < 0.001, Table 1). The LAD occlusion rate was significantly higher in the LVEF improvement group than in the non-improvement group (86.1%±23.7% vs. 70.2%±34.8%, *p* = 0.011, Table 1). No significant differences were observed between the two groups regarding smoking status, hypertension, history of coronary heart disease, time from symptom onset to vessel recanalization, initial TIMI flow grade, number of vessels involved, or culprit vessel location (all *p* > 0.05, Table 1). All patients achieved TIMI 3 flow after PCI.

**Table 1:** Baseline characteristics of patients with STEMI

Baseline characteristics	LVEF non-improvement group (*n* = 68)	LVEF improvement group (*n* = 28)	*P*
Sex, n (%)			0.645
Male	53 (77.9)	23 (82.1)	
Female	15 (22.1)	5 (17.9)	
Age, years	60.46 ± 12.46	59.04 ± 12.87	0.616
LVEF, %	60.25 ± 5.81	48.21 ± 7.83	< 0.001
cTNI, ng/mL	14.29 ± 20.45	21.61 ± 19.33	0.113
Myoglobin, µg/L	266.79 ± 532.34	277.18 ± 369.71	0.928
CK-MB, µg/L	50.14 ± 74.76	99.04 ± 156.47	0.131
BNP, pg/mL	472.80 ± 858.66	1820.40 ± 4492.84	0.134
Creatinine, µmol/L	81.63 ± 31.95	84.22 ± 21.00	0.699
Uric acid, mmol/L	394.62 ± 104.59	375.81 ± 110.91	0.441
Cholesterol, mmol/L	4.52 ± 1.08	4.69 ± 1.36	0.550
Triglycerides, mmol/L	1.89 ± 1.63	1.89 ± 0.93	0.983
LDL-C, mmol/L	2.84 ± 0.87	2.99 ± 1.23	0.512
ALT, U/L	49.34 ± 79.42	38.13 ± 26.24	0.469
AST, U/L	90.98 ± 123.04	111.51 ± 109.58	0.451
D-dimer, mg/L	0.22 ± 0.41	0.33 ± 0.86	0.414
Fibrinogen, g/L	3.24 ± 1.27	3.29 ± 2.05	0.900
White blood cell count	8.47 ± 2.84	8.77 ± 3.20	0.665
Platelet count	225.94 ± 101.92	215.04 ± 60.78	0.618
hs-CRP, mg/L	14.11 ± 22.78	15.50 ± 21.77	0.829
Smoking, n (%)	30 (44.1)	10 (35.7)	0.448
Hypertension, n (%)	34 (50)	13 (46.4)	0.750
Diabetes, n (%)	12 (17.6)	11 (39.3)	0.024
Coronary heart disease, n (%)	9 (13.2)	2 (7.1)	0.500
Time from symptom onset to vessel recanalization, hours	9.94 ± 9.24	10.36 ± 9.14	0.840
Initial TIMI, n (%)			0.184
0	32 (47.1)	19 (67.9)	-
1	9 (13.2)	4 (14.3)	-
2	16 (23.5)	2 (7.1)	-
3	11 (16.2)	3 (10.7)	-
Post-PCI TIMI, n (%)			
3	68 (100)	28 (100)	-
Number of vessels involved, n (%)			0.074
1	40 (58.8)	12 (42.9)	-
2	21 (30.9)	8 (28.6)	-
3	7 (10.3)	8 (28.6)	-
Culprit vessel location, n (%)			0.499
LAD	33 (48.5)	16 (57.1)	-
LCX	14 (20.6)	3 (10.7)	-
RCA	21 (30.9)	9 (32.1)	-
LAD occlusion rate	70.2%±34.8%	86.1%±23.7%	0.011
LCX occlusion rate	49.2%±37.8%	52.7%±33.3%	0.670
RCA occlusion rate	48.7%±41.1%	61.7%±38.1%	0.152

*Abbreviations*: *LVEF* left ventricular ejection fraction, *cTNI* cardiac troponin I, *CK-MB* creatine kinase MB, *BNP* brain natriuretic peptide, *LDL-C* low-density lipoprotein cholesterol, *ALT* alanine aminotransferase, *AST* aspartate aminotransferase, *hs-CRP* hypersensitive C-reactive protein, *TIMI* thrombolysis in myocardial infarction, *LAD* left anterior descending artery, *LCX* left circumflex artery, *RCA* right coronary artery

### Heart size of patients

There was no statistically significant difference in heart size between the LVEF improvement group and the LVEF non-improvement group at 24 h after PCI (all *p* > 0.05, Table 2).

**Table 2:** Heart size in at 24 h after PCI in patients with STEMI

Characteristics	LVEF non-improvement group (*n* = 68)	LVEF improvement group (*n* = 28)	*P*
Ascending aortic internal diameter, mm	29.19 ± 2.63	29.43 ± 3.02	0.706
Left atrial internal diameter, mm	33.34 ± 3.32	35.18 ± 5.24	0.095
Left ventricular end-diastolic diameter, mm	45.82 ± 5.13	49.00 ± 7.94	0.058
Interventricular septum thickness, mm	10.73 ± 1.33	11.00 ± 1.63	0.405
Left ventricular posterior wall thickness in diastole, mm	10.42 ± 1.27	10.59 ± 1.47	0.566
Right atrial diameter, mm	35.69 ± 2.94	35.04 ± 4.25	0.388
Right ventricular diameter, mm	34.33 ± 2.74	33.54 ± 4.05	0.349

*Abbreviations*: *LVEF* left ventricular ejection fraction

### Incidence of MACE

The incidence of MACE was 3.57% in the LVEF improvement group and 7.35% in the LVEF non-improvement group. There was no statistically significant difference in the incidence of MACE between the two groups (*p* = 0.668, Table 3).

**Table 3:** Comparison on incidence of MACE between two groups

MACE	LVEF non-improvement group (*n* = 68)	LVEF improvement group (*n* = 28)	*P*
Cardiac death	0	0	0.668
Myocardial reinfarction	4	1	
Acute heart failure	1	0	
Malignant arrhythmia	0	0	
Overall incidence	7.35%	3.57%	

*Abbreviations*: *MACE* major adverse clinical events, *LVEF* left ventricular ejection fraction

### Serum TWEAK, TRAIL, and soluble Dectin-1 Levels

**Fig. 1:**
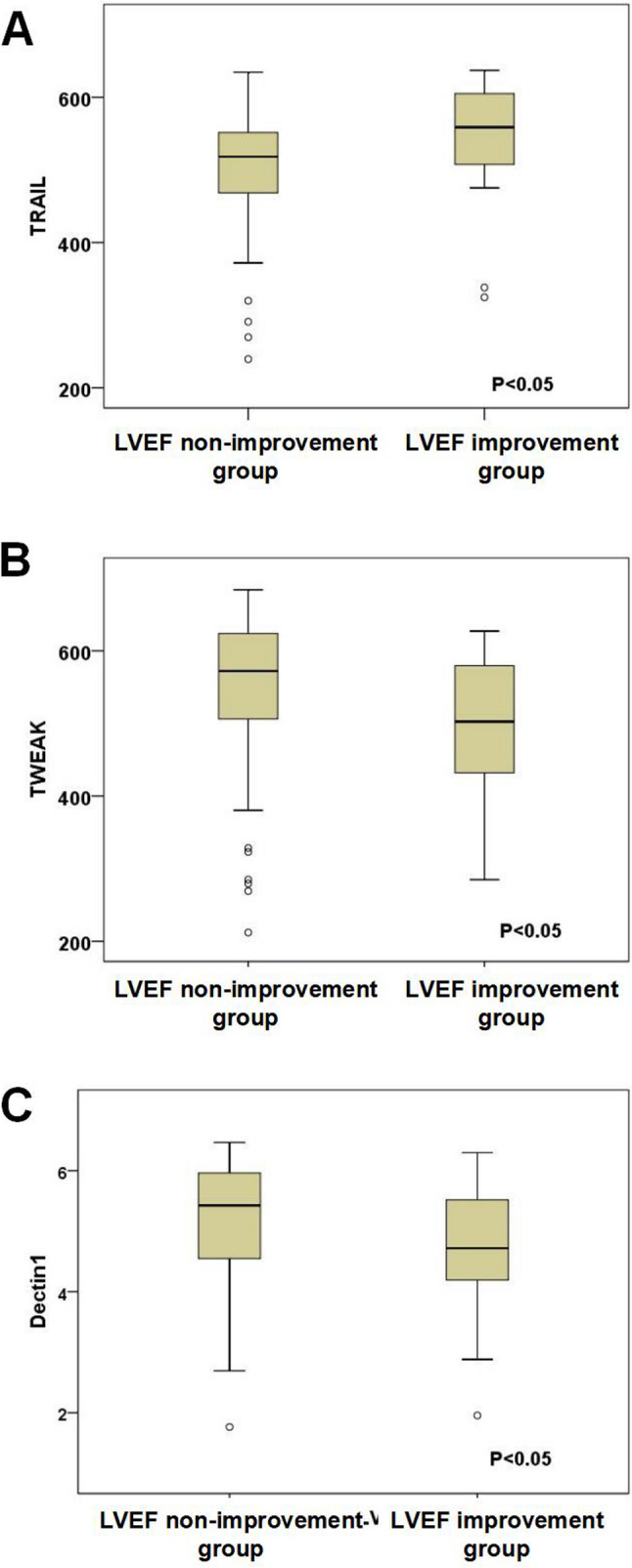
Comparison of serum TRAIL (**A**), TWEAK (**B)**, and soluble Dectin-1 levels (**C**) at admission between patients in the LVEF improvement group and those in the LVEF non-improvement group

The patients in the LVEF improvement group showed significantly higher TRAIL level upon admission (545.60 ± 78.63 pg/mL vs. 501.54 ± 82.52 pg/mL; *p* < 0.05; Fig. 1A) compared with those in the LVEF non-improvement group. Nevertheless, the TWEAK (490.5 ± 0.5 pg/mL vs. 542.71 ± 111.60 pg/mL; *p* < 0.05; Fig. 1B) and soluble Dectin-1 (4.69 ± 1.08 pg/mL vs. 5.18 ± 0.97 pg/mL; *p* < 0.05; Fig. 1C) in the LVEF improvement group were significantly lower compared with those in the LVEF non-improvement group.

Multivariable logistic regression analysis adjusting for age, sex, diabetes, and LAD occlusion rate showed that TRAIL (*P* = 0.047) and TWEAK (*P* = 0.005) were independently associated with LVEF improvement (Table 4). Among the 96 patients, 80 (83.3%) had baseline LVEF ≥ 50% and 16 (16.7%) had baseline LVEF < 50%. In the LVEF non-improvement group, 65 patients (95.6%) had baseline LVEF ≥ 50% and only 3 (4.4%) had baseline LVEF < 50%. In the LVEF improvement group, 13 patients (46.4%) had baseline LVEF ≥ 50% and 15 (53.6%) had baseline LVEF < 50%. To address the concern of regression-to-the-mean, a subgroup analysis was performed in patients with baseline LVEF ≥ 50%. In this subgroup, multivariable logistic regression analysis showed that TWEAK remained a significant predictor of LVEF improvement (OR = 0.992, 95% CI: 0.985-1.000, *P* = 0.047, Table 5).

**Table 4:** Multivariable logistic regression analysis in total population

Variables	Univariate Analysis		Multivariate analysis	
	OR (95% CI)	*p*	OR (95% CI)	*p*
Dectin-1	1.625 (0.995, 2.654)	0.052	1.467 (0.645, 3.337)	0.360
TRAIL	1.008 (1.001, 1.015)	0.023	1.011 (1.000, 1.021)	0.047
TWEAK	0.996 (0.992, 1.000)	0.039	0.991 (0.985, 0.997)	0.005
Age	0.991 (0.957, 1.026)	0.612	0.989 (0.949, 1.032)	0.616
Sex (Male vs. Female)	1.302 (0.423, 4.007)	0.646	0.813 (0.178, 3.712)	0.789
Diabetes (Yes vs. No)	3.020 (1.131, 8.060)	0.027	2.564 (0.771, 8.527)	0.125
LAD occlusion rate	1.019 (1.001, 1.038)	0.037	1.012 (0.993, 1.032)	0.222

*Abbreviations*: *Dectin-1* dendritic cell-associated C-type lectin-1, *TRAIL* tumor necrosis factor-related apoptosis-inducing ligand, *TWEAK* tumor necrosis factor-like weak inducer of apoptosis, *OR* odds ratio, *CI* confidence interval, *LAD* left anterior descending artery

**Table 5:** Multivariable logistic regression analysis in patients with LVEF ≥ 50%

Variables	Univariate Analysis		Multivariate analysis	
	OR (95% CI)	*p*	OR (95% CI)	*p*
Dectin-1	1.644 (0.874, 3.090)	0.123	1.643 (0.564, 4.787)	0.363
TRAIL	1.007 (0.998, 1.016)	0.106	1.010 (0.996, 1.025)	0.153
TWEAK	0.996 (0.992, 1.001)	0.130	0.992 (0.985, 1.000)	0.047
Age	0.985 (0.943, 1.028)	0.480	0.981 (0.932, 1.033)	0.474
Sex (Male vs. Female)	1.200 (0.299, 4.820)	0.797	0.721 (0.101, 5.142)	0.744
Diabetes (Yes vs. No)	3.273 (0.967, 11.081)	0.057	3.294 (0.687, 15.800)	0.136
LAD occlusion rate	1.025 (0.998, 1.052)	0.066	1.020 (0.991, 1.049)	0.179

*Abbreviations*: *Dectin-1* dendritic cell-associated C-type lectin-1, *TRAIL* tumor necrosis factor-related apoptosis-inducing ligand, *TWEAK* tumor necrosis factor-like weak inducer of apoptosis, *OR* odds ratio, *CI* confidence interval, *LVEF* left ventricular ejection fraction, *LAD* left anterior descending artery

### Correlation among serum TWEAK, TRAIL, and soluble Dectin-1 levels

**Fig. 2:**
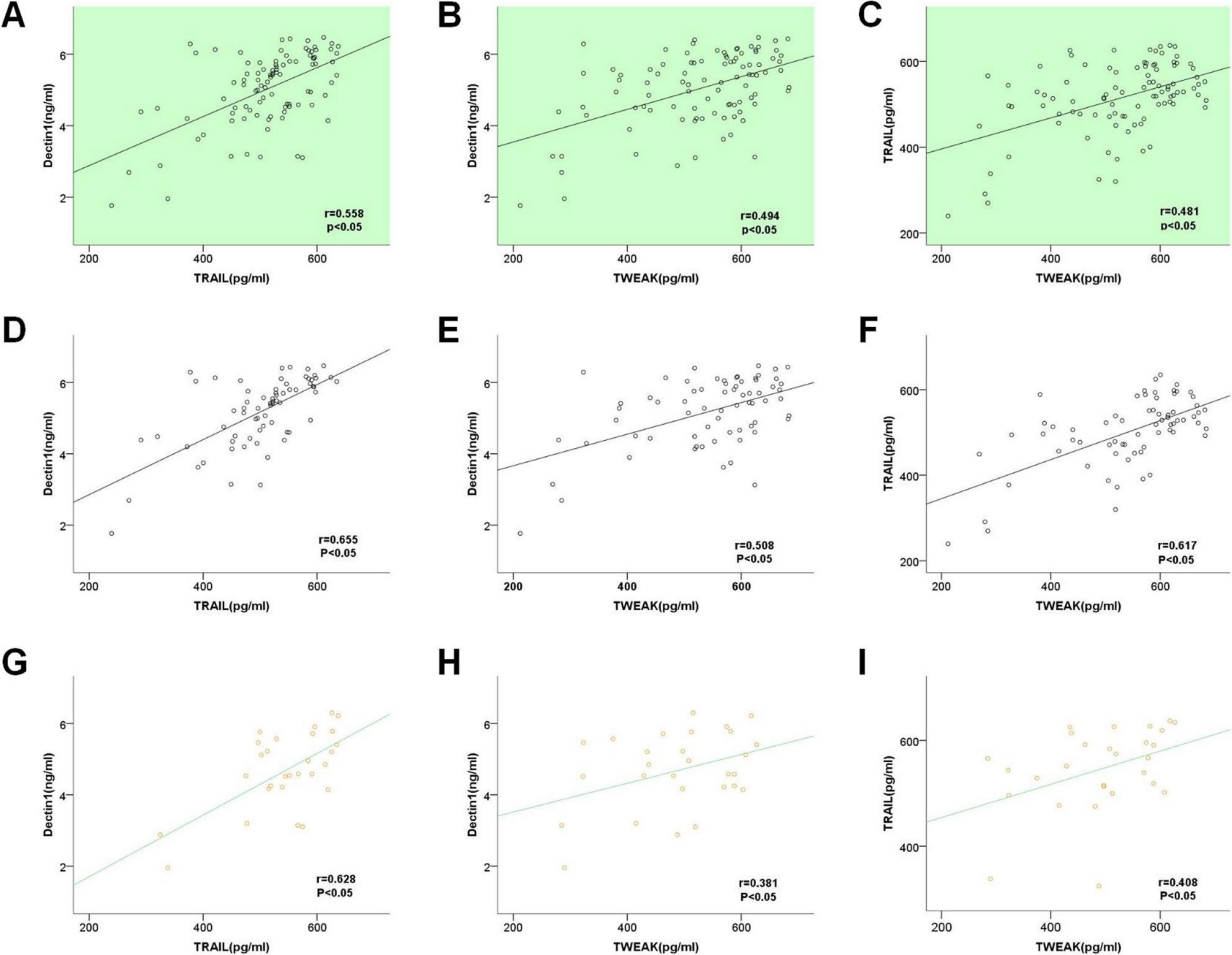
Correlation of serum soluble Dectin-1 levels, TRAIL, and TWEAK in all patients (**A**-**C**), in patients of the LVEF improvement group (**D**-**F**), and patients of the LVEF non-improvement group (**G**-**I**)

There were significant correlations among serum levels of soluble Dectin-1, TRAIL, and TWEAK in all patients (*r* = 0.558, *r* = 0.494, *r* = 0.481, respectively, all *P* < 0.05; Fig. 2A, B and C), the LVEF improvement group (*r* = 0.655, *r* = 0.508, *r* = 0.617, respectively, all *P* < 0.05; Fig. 2D, E and F) and the non-improvement group (*r* = 0.628, *r* = 0.381, *r* = 0.408, respectively, all *P* < 0.05; Fig. 2G, H and I).

### ROC curves of serum TRAIL, soluble Dectin-1, and TWEAK levels to LVEF improvement

**Fig. 3:**
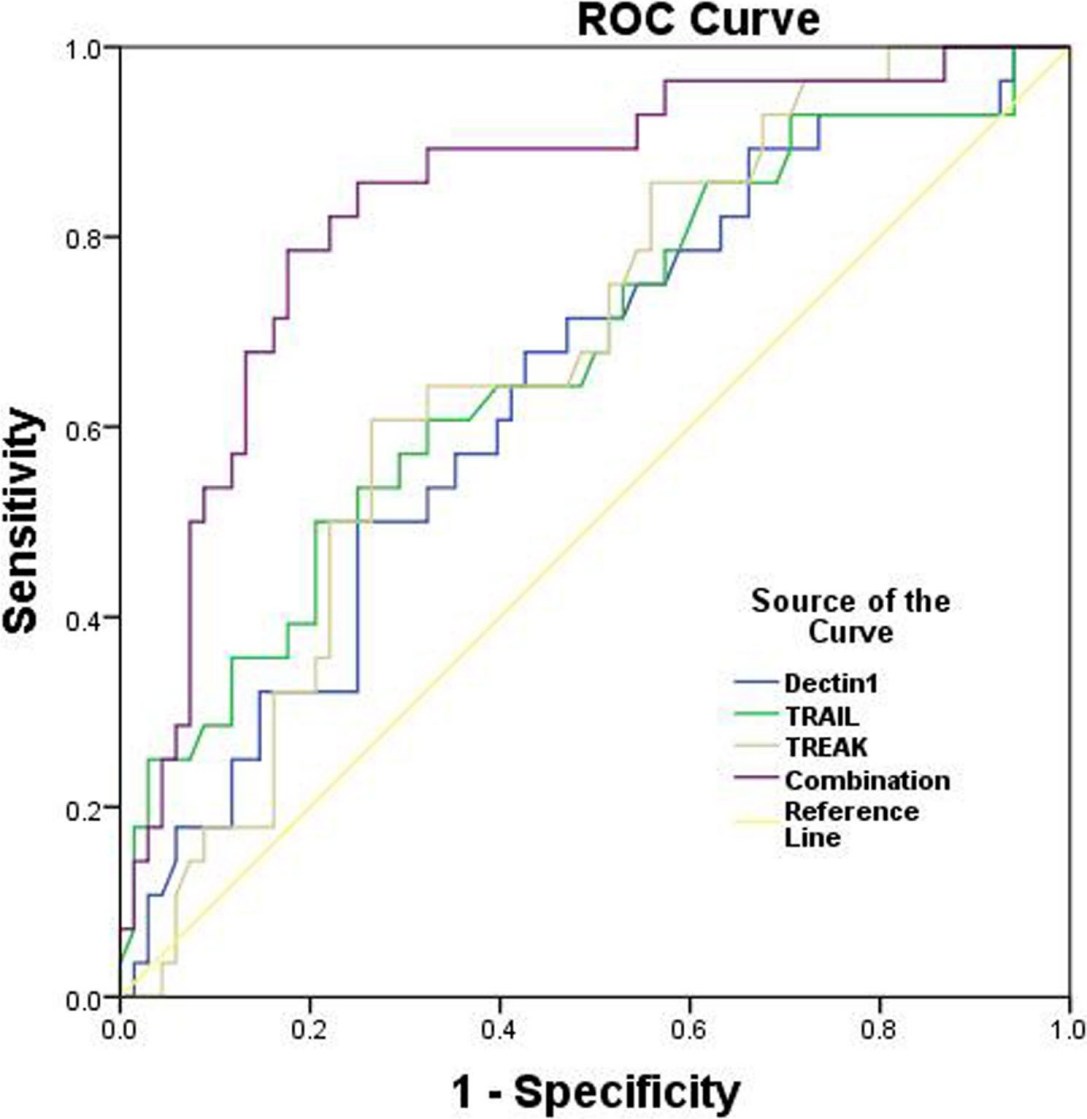
ROC curves of serum TWEAK, TRAIL, soluble Dectin-1 levels, and the combination of three biomarkers to predict LVEF improvement

For serum TRAIL, the AUC was 0.676 (95% confidence interval [CI]: 0.56–0.80, *p* < 0.05), with a critical value of 564.45 pg/mL, sensitivity of 50.00%, and specificity of 79.41%. Serum soluble Dectin-1 levels yielded an AUC of 0.642 (95% CI: 0.52–0.76, *P* < 0.05), with a critical value of 5.25 ng/mL, sensitivity of 67.86%, and specificity of 57.35%. Serum TWEAK exhibited an AUC of 0.671 (95% CI: 0.56–0.78, *P* < 0.05), with a critical value of 516.38 pg/mL, sensitivity of 60.71%, and specificity of 73.53%. Remarkably, the AUC, sensitivity, and specificity of combining all three biomarkers were 0.838 (95% CI: 0.748–0.927, *P* < 0.05), 78.57%, 82.35%, respectively (Fig. 3).

## Discussion

To the best of our knowledge, this study is among the first to explore the prediction of post-PCI LVEF improvement in patients with STEMI using apoptotic markers. The results suggested that serum levels of TWEAK, TRAIL, and soluble Dectin-1 may serve as potential predictive biomarkers for LVEF improvement in patients with STEMI after PCI. The combination of the three biomarkers showed improved prediction accuracy, sensitivity, and specificity.

Cardiomyocyte apoptosis plays a pivotal role in ischemic cardiomyocyte death and development of heart failure. Autopsy findings from patients with AMI demonstrated biochemical features which were consistent with cardiomyocyte apoptosis, while animal studies illustrated its contribution to ischemic cardiomyocyte necrosis, infarct expansion, ventricular remodeling, and subsequent heart failure [4, 5]. By evaluating TWEAK, TRAIL, and soluble Dectin-1, we intended to provide valuable insights into post-infarction cardiac function and to refine prognostic strategies for individuals recovering from AMI.

The prediction model in this study focused on the prediction of improvement of LVEF rather than major adverse cardiovascular events [15, 16], in-hospital mortality [17], or intramyocardial hemorrhage [19]. In a previous study of predicting left ventricular systolic dysfunction at six months after STEMI, only clinical parameters, including baseline and post-PCI ECG parameters, were considered in the model [18], while apoptotic biomarkers were not included. In the current study, the combination of TWEAK, TRAIL, and soluble Dectin-1 showed potential as a prediction model for LVEF improvement in patients with STEMI after PCI.

A negative correlation between serum TWEAK level and the improvement of LVEF in patients with STEMI after PCI was indicated in this study. TWEAK directly inhibits the expression of peroxisome proliferator-activated receptor gamma coactivator 1α, a critical regulator of cardiomyocyte metabolism and oxidative phosphorylation genes [20], which exacerbates left ventricular dysfunction following infarction. Patients with AMI have significantly higher serum levels of TWEAK compared to healthy subjects and patients with stable coronary artery disease [21]. Furthermore, elevated serum TWEAK levels upon admission were associated with increased short-term adverse outcomes in patients with STEMI [22].

In this study, patients with STEMI undergoing PCI exhibited a relationship between lower TRAIL levels and reduced LVEF, which were consistent with previous study by Teringova et al. [23]. Similarly, Osmancik et al. highlighted serum TRAIL level as a robust predictor of prognosis in acute coronary syndromes, with lower levels associated with poorer outcomes [24]. The results of our study further suggested that apoptosis markers also held prognostic value in coronary heart diseases such as STEMI.

Notably, this study observed an intriguing pattern in which TRAIL levels were higher while TWEAK levels were lower in the LVEF improvement group, which was characterized by larger myocardial infarct sizes. Despite both belonging to the TNF superfamily and possessing the ability to induce necroptosis, these two molecules appear to engage fundamentally divergent signaling pathways in the cardiac context. TRAIL, through its death receptor DR5, predominantly activates non-canonical pro-survival signaling cascades in cardiovascular cells, including EGFR transactivation and downstream ERK1/2 signaling that promotes adaptive cardiomyocyte hypertrophy [25], as well as Akt and ERK1/2 activation that promotes endothelial cell survival and proliferation [26]. Furthermore, TRAIL/DR5 signaling selectively induces apoptosis in activated myofibroblasts, thereby suppressing maladaptive fibrotic remodeling [27]. In contrast, the TWEAK/Fn14 axis predominantly activates NF-κB pathways, suppresses PGC-1α expression, and promotes pro-inflammatory macrophage infiltration in the post-infarction myocardium [20]. Additionally, TWEAK is cleared from the circulation by the macrophage scavenger receptor CD163, and lower circulating TWEAK levels in patients with favorable recovery may reflect an effective anti-inflammatory macrophage response [28]. Thus, the combination of elevated TRAIL and decreased TWEAK may represent a favorable immunological and reparative profile that facilitates functional recovery after myocardial infarction.

This study showed lower soluble Dectin-1 levels in the LVEF improvement group compared to that in the LVEF non-improvement group. Studies have shown that Dectin-1 expression is upregulated in the early stages of heart ischemia-reperfusion, with evidence from cell and animal experiments suggesting its involvement in cardiomyocyte apoptosis, exacerbating myocardial ischemia-reperfusion injury, and worsening cardiac function. Additionally, Dectin-1 promotes macrophage polarization towards pro-inflammatory M1 macrophages and facilitates the infiltration of Ly6C^+^ monocytes and centroblasts. Elevated Dectin-1^+^ monocytes in the blood of patients with AMI who received PCI treatment compared to those with normal contrast underscores the close association between Dectin-1^+^ cells, AMI, and post-infarction cardiac insufficiency [14].

In this study, the incidence of MACE in the LVEF non-improvement group was numerically higher compared to that in the LVEF improvement group without statistical significance, which was in line with a previous study by Kyung An Kim et al. [29]. Nevertheless, patients with persistently reduced LVEF still had a higher risk of cardiovascular mortality and rehospitalization due to heart failure than those with normal LVEF at baseline [30].

This study has several limitations. First, the sample size was relatively small due to the COVID-19 epidemic. Second, several clinical variables were not available for adjustment due to limited data collection conditions. Third, the predictive model was developed and tested in the same cohort without external validation, and the risk of overfitting cannot be excluded given the small sample size. We are currently constructing a prospective study in an external cohort to validate the preliminary findings from the current study. Fourth, the underlying downstream mechanisms responsible for the opposite expression trends of TWEAK and TRAIL in the context of LVEF improvement remain unclear and warrant further investigation. Further studies with larger sample sizes and prospective validation are needed to ascertain the predictive value of the combination of serum TRAIL, TWEAK, and soluble Dectin-1 levels in LVEF improvement in post-PCI patients with STEMI.

## Conclusions

In conclusion, in this retrospective study, post-PCI patients with STEMI showed higher TRAIL levels, and lower TWEAK and soluble Dectin-1 levels in the LVEF improvement group compared to the LVEF non-improvement group. TRAIL, TWEAK, and soluble Dectin-1 levels may serve as potential predictors of post-PCI LVEF improvement in patients with STEMI, and the combination of these three biomarkers showed improved prediction accuracy. These preliminary findings suggest that these biomarkers may have potential utility in assessing cardiac function recovery in post-PCI patients with STEMI, though further validation in larger, prospective cohorts is needed.

## Data Availability

The datasets used and/or analysed during the current study are available from the corresponding author on reasonable request.

## Abbreviations

AMI: Acute myocardial infarction
PCI: Percutaneous coronary intervention
STEMI: ST-elevation myocardial infarction
TWEAK: Tumor necrosis factor-like weak inducer of apoptosis
TRAIL: Tumor necrosis factor-related apoptosis-inducing ligand
Dectin-1: Dendritic cell-associated C-type lectin-1
LVEF: Left ventricular ejection fraction
CAG: Coronary angiography
ELISA: Enzyme-linked immunosorbent assay
MACE: Major adverse cardiovascular events
ROC: Receiver operating characteristic
AUC: Area under the curves
CI: Confidence interval
